# Supplementary material for: Cdk5 drives formation of heterogeneous pancreatic neuroendocrine tumors
Source: Oncogenesis. 2021 Dec 3;10(12):83. doi: 10.1038/s41389-021-00372-5 (PMC8642406; doi:10.1038/s41389-021-00372-5)
Supplement: Supplementary file 1 — Supplemental material [file 41389_2021_372_MOESM1_ESM.pdf]

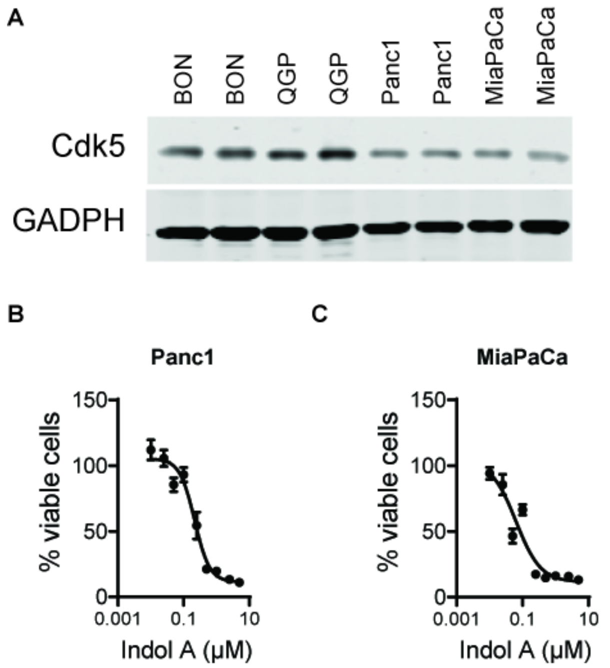

Supplemental Figure S1. Inhibition of Cdk5 blocks growth of PDAC cell lines. A. Immunoblot of Cdk5 and GAPDH in PDAC cells. B-C. PDAC cell lines were treated with increasing concentrations of Indo A and monitored for effects on cell viability. Error bars represent SEM.

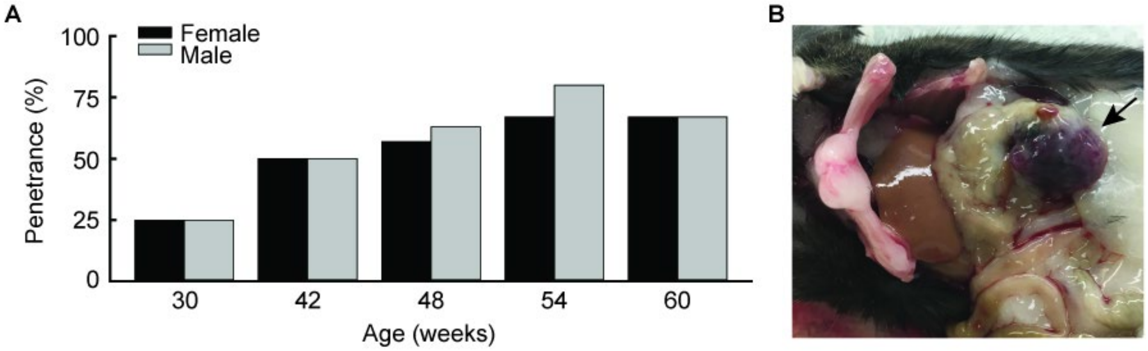

Supplemental Figure S2. Penetrance of PanNETs in INS-p25OE model. A. Detection of visible tumors by autopsy (n=4-8). B. Representative image of PanNET in autopsy; tumor marked with black arrow.

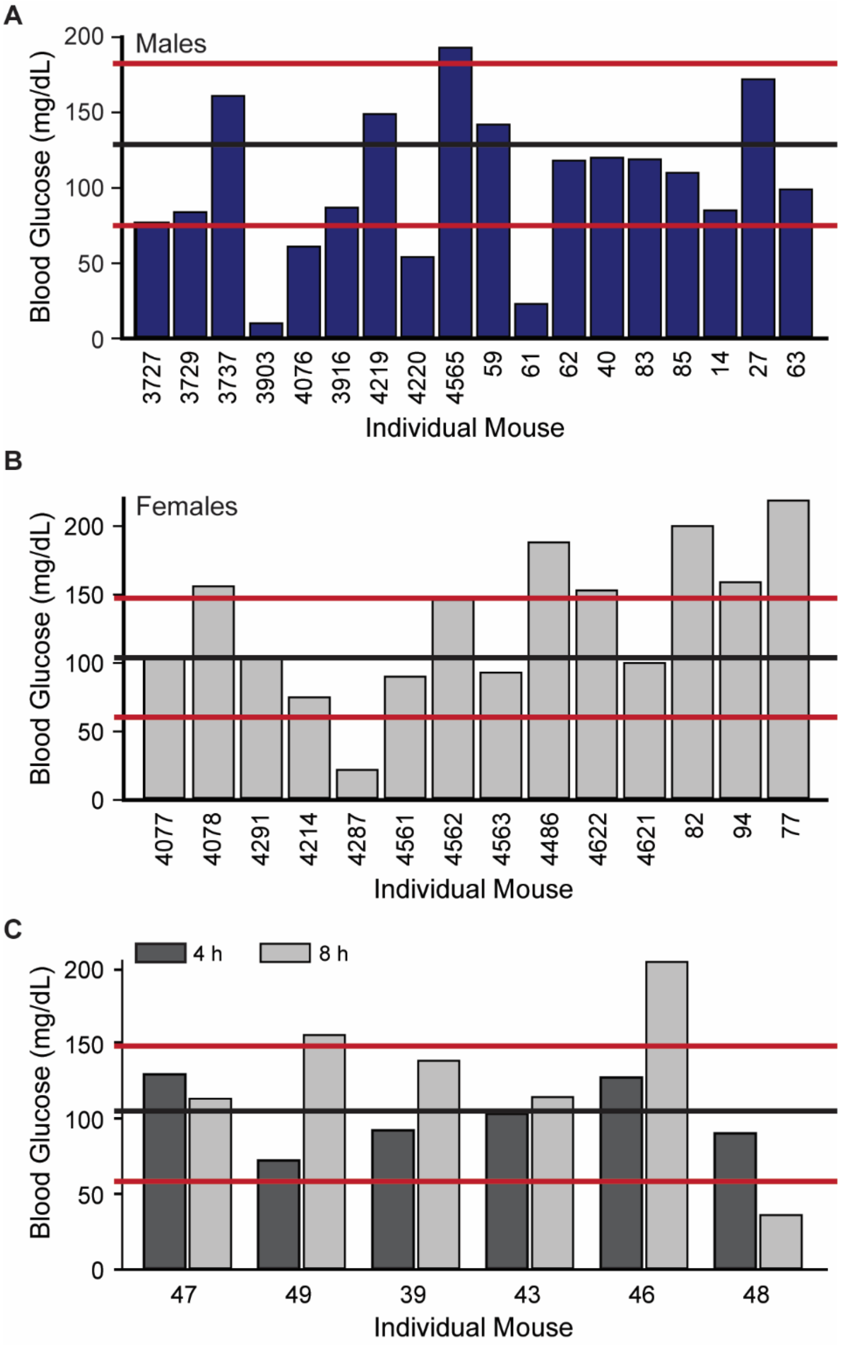

Supplemental Figure S3. INS-p25OE animals exhibit low penetrance of hypoglycemia. A-B. Blood glucose levels in male (A) and female (B) INS-p25OE tumor-bearing animals (n=18 and 14, respectively) after 4 h fast. C. Blood glucose levels in female INS-p25OE tumor-bearing animals after 4 and 8 h fast (n=6). Black lines represent average glucose levels in control littermates. Red lines denote two standard deviations from average of controls.

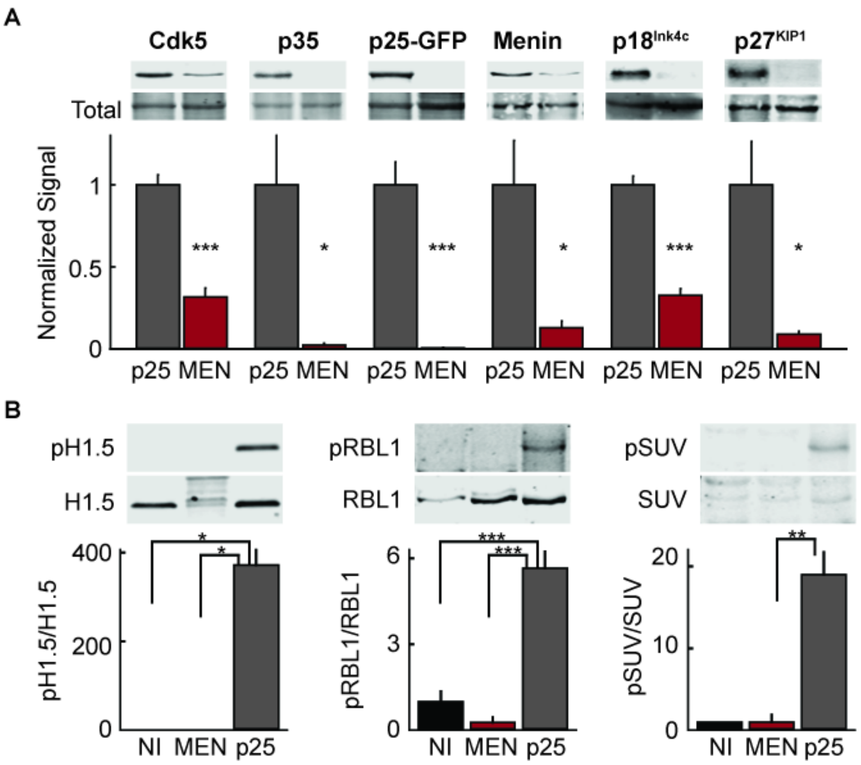

Supplemental Figure S4. Cdk5 and menin pathways are distinct drivers of mPanNETs. A. Quantitative immunoblot of Cdk5 pathway components, menin, and downstream targets of menin in $MEN^{+/-}$ tumors (MEN; n=4) and INS-p25OE tumors (p25; n=5). B. Quantitative immunoblot of downstream targets of Cdk5 in normal mouse islets (NI; n=3), $MEN^{+/-}$ tumors (MEN; n=4), and INS-p25OE tumors (p25; n=7); phosphorylated Ser18-H1.5 (pH1.5), phosphorylated Ser988-RBL1 (pRBL1), phosphorylated Ser391-SUV39H1 (pSUV).

A

Sample	Size (n)	Accession #
Mouse PanNETs	6	GSE176448_T
Human PanNETs	17	GSE116356
Human PanNETs	113	GSE98894
Human PDACs	51	GSE79668

B

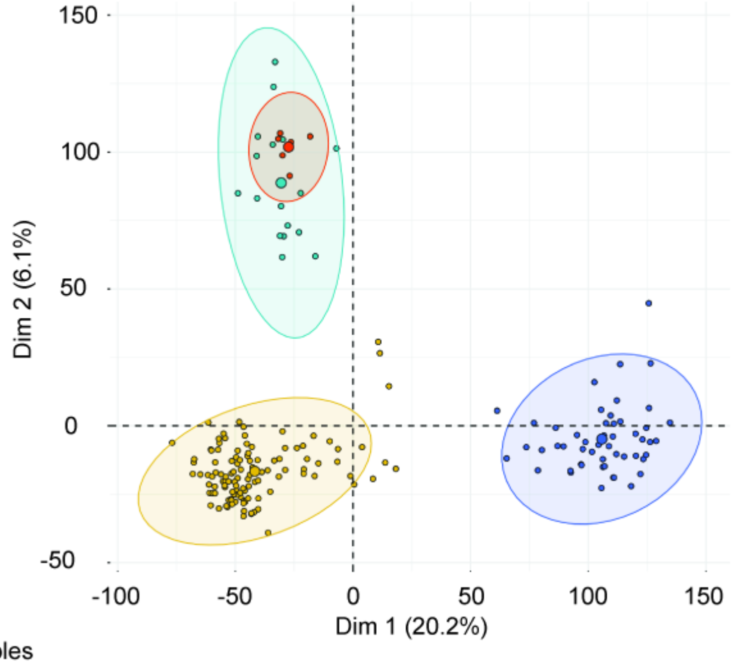

C

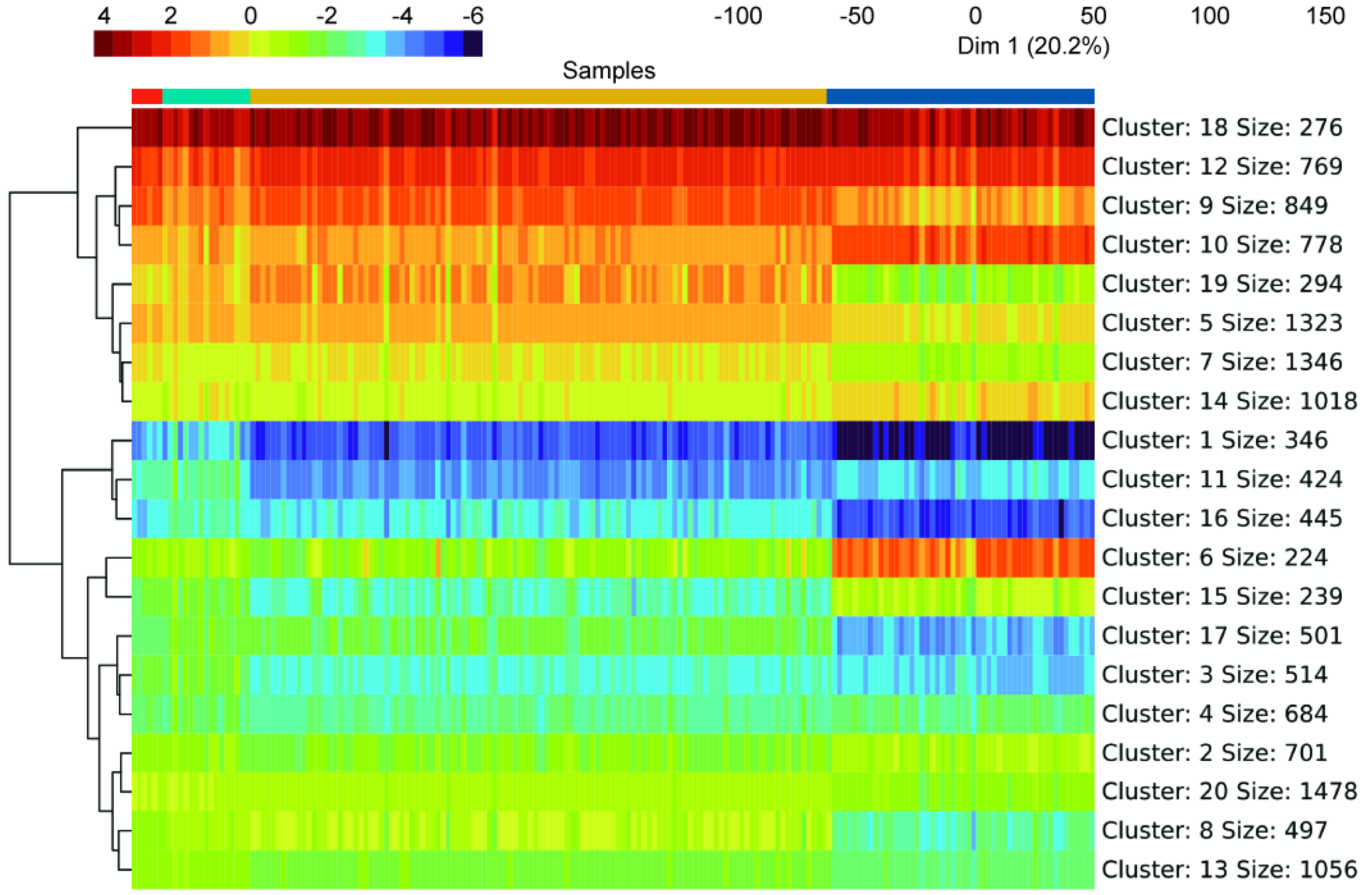

Supplemental Figure S5. INS-p25OE tumors and human PanNETs exhibit similar overall gene expression patterns. A. Chart of sample sets analyzed. B. Principal component analysis of sample sets. C. Heat map illustrating expression levels in clusters of genes with similar expression profiles; 20 genes in each cluster. Clusters determined by K-means clustering algorithm.

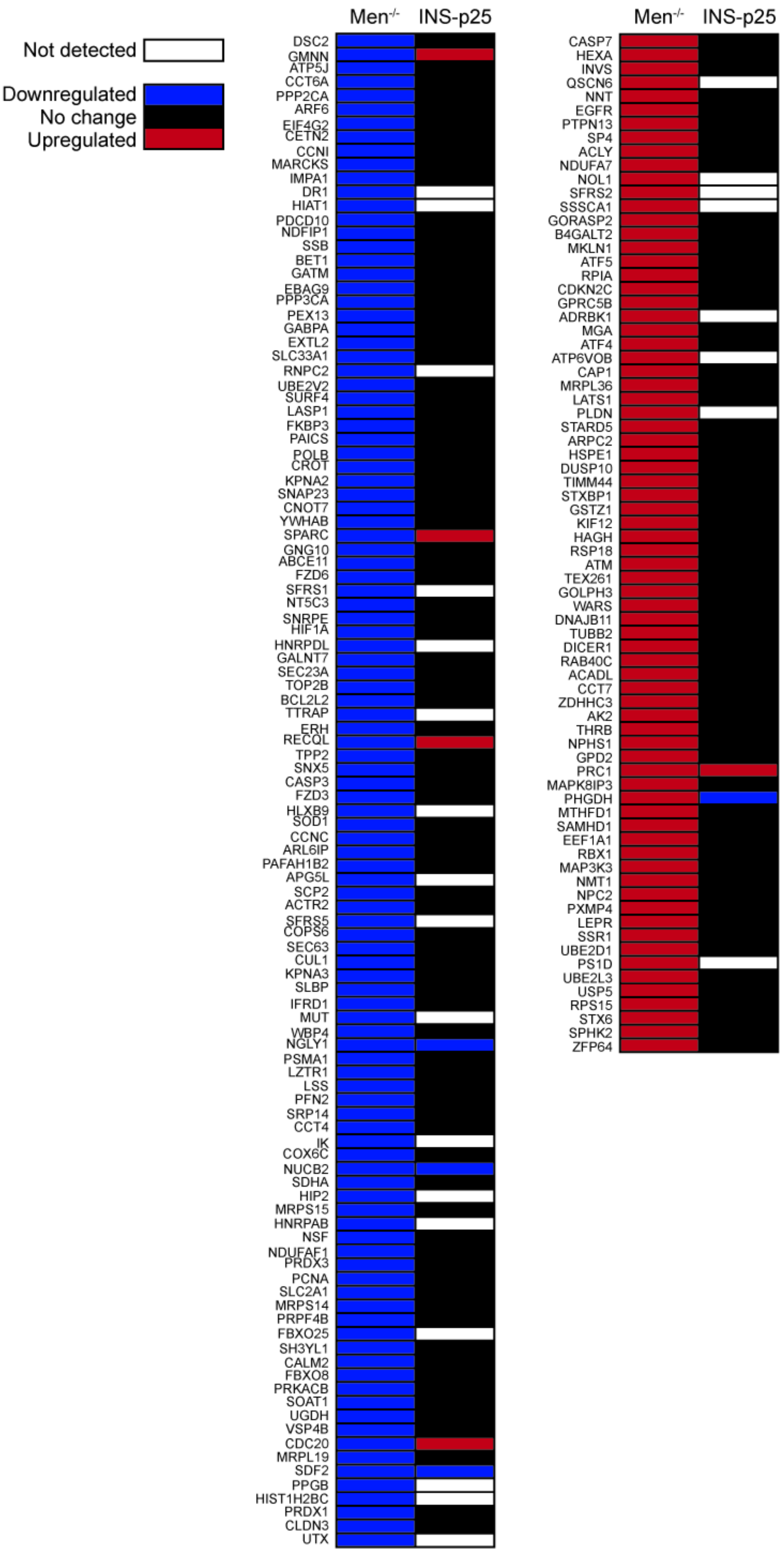
Not detected
Downregulated
No change
Upregulated
Men-/-
INS-p25
DSC2
GMNN
ATP5J
CCT6A
PPP2CA
ARF6
EIF4G2
CETN2
CCNI
MARCKS
IMPA1
DR1
HIAT1
PDCD10
NDFIP1
SSB
BET1
GATM
EBAG9
PPP3CA
PEX13
GABPA
EXTL2
SLC33A1
RNPC2
UBE2V2
SURF4
LASP1
FKBP3
PAICS
POLB
CROT
KPNA2
SNAP23
CNOT7
YWHAB
SPARC
GNG10
ABCE11
FZD6
SFRS1
NT5C3
SNRPE
HIF1A
HNRPDL
GALNT7
SEC23A
TOP2B
BCL2L2
TTRAP
ERH
RECQL
TPP2
SNX5
CASP3
FZD3
HLXB9
SOD1
CCNC
ARL6IP
PAFAH1B2
APG5L
SCP2
ACTR2
SFRS5
COPS6
SEC63
CUL1
KPNA3
SLBP
IFRD1
MUT
WBP4
NGLY1
PSMA1
LZTR1
LSS
PFN2
SRP14
CCT4
IK
COX6C
NUCB2
SDHA
HIP2
MRPS15
HNRPAB
NSF
NDUFAF1
PRDX3
PCNA
SLC2A1
MRPS14
PRPF4B
FBXO25
SH3YL1
CALM2
FBXO8
PRKACB
SOAT1
UGDH
VSP4B
CDC20
MRPL19
SDF2
PPGB
HIST1H2BC
PRDX1
CLDN3
UTX
Men-/-
INS-p25
CASP7
HEXA
INVS
QSCN6
NNT
EGFR
PTPN13
SP4
ACLY
NDUFA7
NOL1
SFRS2
SSSCA1
GORASP2
B4GALT2
MKLN1
ATF5
RPIA
CDKN2C
GPRC5B
ADRBK1
MGA
ATF4
ATP6VOB
CAP1
MRPL36
LATS1
PLDN
STARD5
ARPC2
HSPE1
DUSP10
TIMM44
STXBP1
GSTZ1
KIF12
HAGH
RSP18
ATM
TEX261
GOLPH3
WARS
DNAJB11
TUBB2
DICER1
RAB40C
ACADL
CCT7
ZDHHC3
AK2
THRB
NPHS1
GPD2
PRC1
MAPK8IP3
PHGDH
MTHFD1
SAMHD1
EEF1A1
RBX1
MAP3K3
NMT1
NPC2
PXMP4
LEPR
SSR1
UBE2D1
PS1D
UBE2L3
USP5
RPS15
STX6
SPHK2
ZFP64

Supplemental Figure S6. Cdk5 and menin pathways alter disparate transcriptional profiles. Heat map of genes found to be differentially expressed in men$^{-/-}$ islets compared to normal islets, by prior microarray analysis (184 total)[51], and the corresponding differential expression in INS-p25OE tumors compared to normal islets, detected by RNAseq.

Supplemental Table S1. Map of human PanNET TMA.

Normal Spleen	WD-NET NF Grade 1	WD-NET NF Grade 1	WD-NET NF Grade 1	WD-NET NF Grade 2	WD-NET NF Grade 1	WD-NET NF Grade 1	WD-NET NF Grade 2	WD-NET NF Grade 1
Normal Liver	WD-NET NF Grade 1	WD-NET NF Grade 2	WD-NET NF Grade 2	WD-NET NF Grade 2	WD-NET NF Grade 2	WD-NET NF Grade 2	WD-NET NF Grade 1	WD-NET NF Grade 1
Normal Prostate	WD-NET NF Grade 2	WD-NET NF Grade 1	WD-NET NF Grade 1	WD-NET NF Grade 1	WD-NET NF Grade 2	WD-NET NF Grade 1	WD-NET NF Grade 1	Blank
Normal Placenta	WD-NET NF Grade 1	WD-NET NF Grade 1	WD-NET NF Grade 2	WD-NET NF Grade 1	WD-NET NF Grade 1	WD-NET NF Grade 1	WD-NET Insulinoma Grade 2	WD-NET Insulinoma Grade 1
Normal Tonsil	WD-NET NF Grade 3	Blank	WD-NET NF Grade 2	WD-NET NF Grade 1	WD-NET NF Grade 2	WD-NET NF Grade 1	WD-NET NF Grade 1	Blank

*WD - Well-differentiated, NF – Non-functional

Supplemental Table S2. Mutation frequency, in humans, for genes mutated in INS-p25OE PanNETs.

SYMBOL	GENE_ID	Frequency (%) in Humans
TP53	ENSG00000141510	4
TTN	ENSG00000155657	5
OBSCN	ENSG00000154358	3
FBN2	ENSG00000138829	3
SH3BGR	ENSG00000185437	2
COL18A1	ENSG00000182871	2
NUDCD1	ENSG00000120526	2
RYR2	ENSG00000198626	4
NAT6	ENSG00000243477	2
PTPRQ	ENSG00000139304	2
MMP24	ENSG00000125966	2
NOTCH3	ENSG00000074181	2
NT5DC1	ENSG00000178425	2
OSBPL8	ENSG00000091039	1
ATAD2	ENSG00000156802	1
HEATR5A	ENSG00000129493	1
MLXIPL	ENSG00000009950	1
SH2D3C	ENSG00000095370	1
RBM15B	ENSG00000179837	1
TBC1D9B	ENSG00000197226	1
NFKB2	ENSG00000077150	1
SON	ENSG00000159140	1
MAP4K4	ENSG00000071054	1
SPPL3	ENSG00000157837	1
PML	ENSG00000140464	1
ANKRD17	ENSG00000132466	1
TKTL2	ENSG00000151005	1
KPNA2	ENSG00000182481	1
ROBO2	ENSG00000185008	1
GIGYF1	ENSG00000146830	1
ELK1	ENSG00000126767	1
IPO4	ENSG00000196497	1
OTOF	ENSG00000115155	1
ZC3H18	ENSG00000158545	1
KIF26A	ENSG00000066735	1
ZFHX3	ENSG00000140836	1
SPG7	ENSG00000197912	1
PLXND1	ENSG00000004399	1
SCUBE1	ENSG00000159307	1
MIB2	ENSG00000197530	1
HMCN2	ENSG00000148357	1
NCOR2	ENSG00000196498	1
TRNAU1AP	ENSG00000180098	1
GALNT6	ENSG00000139629	1
MDN1	ENSG00000112159	1
PACSIN3	ENSG00000165912	1
CCDC30	ENSG00000186409	1
DOCK2	ENSG00000134516	1
